# Supplementary figures and images for: A network-based discovery of prognostic markers in recurrent IDH wild-type gliomas
Source: Front Genet. 2025 Nov 20;16:1672015. doi: 10.3389/fgene.2025.1672015 (PMC12675371; doi:10.3389/fgene.2025.1672015)

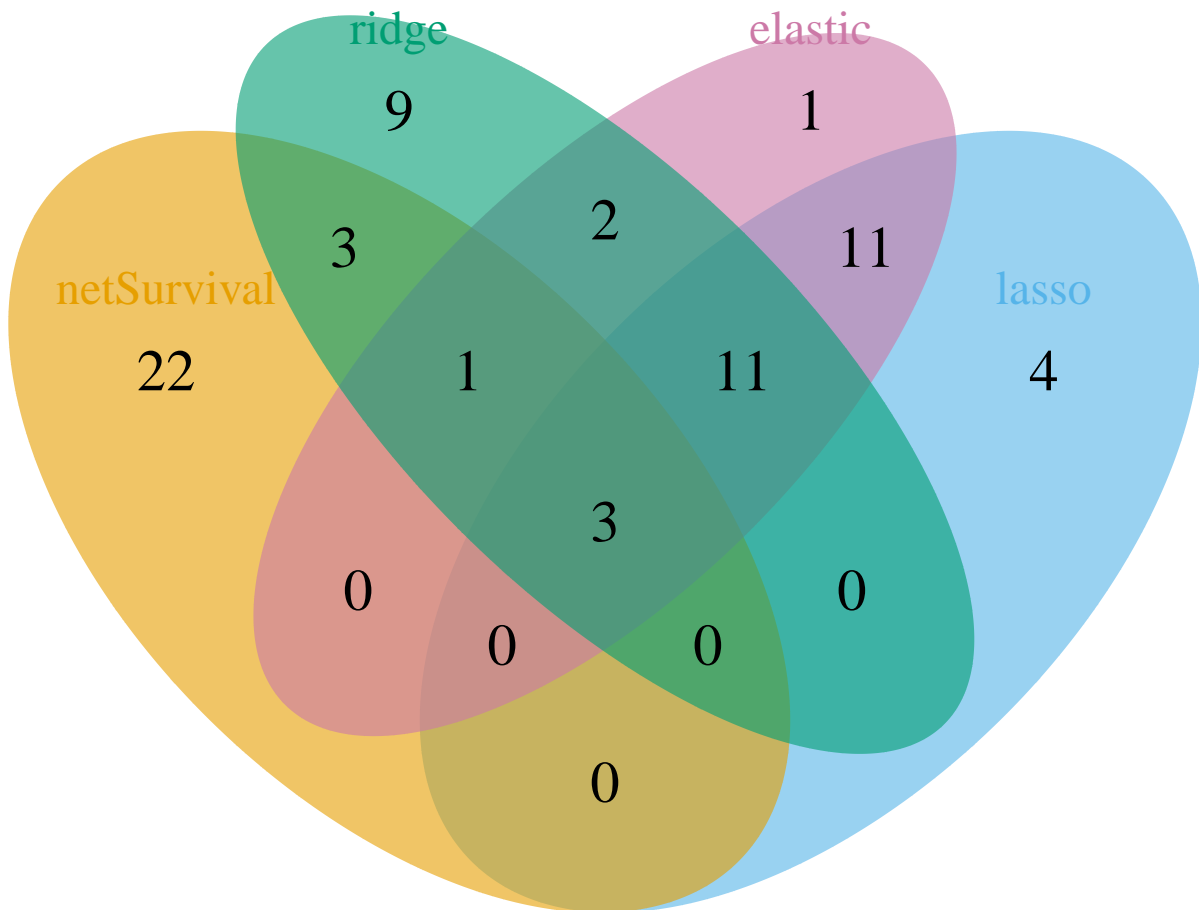

Supplement: Supplementary file 1 [file DataSheet1.pdf]
